# Historical Sketch of the Progress of Medicine in the Year 1808

**Published:** 1809-08

**Authors:** 


### Historical Sketch of the Progress of Medicine in the Year 1808; by Mr. Royston.

( Continued from our last, p. 16. )

ON contagion, as confounded with and substituted for epidemical diseases, having an atmospherical origin; and on the danger of making epidemical constitutions of the air supply the cause of diseases heretofore believed contagious, much has been written since the re-appearance of yellow fever in America, and its extension to Europe, without bringing the question to a rational issue. Dr. Robertson has very properly appropriated a part of his work to this important subject, and his inquiries have induced him to conclude that it has been ascertained,

1st. That no change of the ordinary physical or chemical properties of the atmosphere takes place during the prevalence of pestilential diseases.

2dly. That the occurrence of epidemics is not prevented by the succession of seasons, nor by remarkable changes of weather.

3dly. That although it be admitted, that some of the most malignant species of epidemics arise from causes that are not originally contagious, yet this circumstance appears to be sufficiently explained, as being the consequence of the malignant nature of those causes having propagated a disease, which in its course has generated a matter capable of producing a certain definite train of symptoms at any distance from its original source; and it is owing to this, that it is frequently observed, that the diseases occasioned by contagion do not resemble those by which the contagious matter had been originally produced.

Researches into the causes that act so generally and so extensively on the frames of animals, as to occasion epidemic or contagious fever; and into what peculiarity it is that produces some diseases in particular places only, or in a greater proportion than in others, are among the most difficult and arduous employments of human intellect. Prejudice, self-interest, the ignorance and superstition of former ages, pre-conceived opinions and favorite hypotheses, darken still more the obscurity. Among those who have best laboured to elucidate this important question, as well as the causes of the increase and decrease of diseases, the changes in climate, and the effects of particular states of the weather and seasons on human health; it will be gratifying, to Englishmen at least, to notice the names of Grant, Sharp, Fothergill, and Heberden. To thes may now be added Dr. Woolcombe, who has, in a series of observations drawn from facts and supported by calculations founded on admitted data, endeavoured to ascertain the frequency and fatality of several diseases, particularly the progressive increase of consumption, and the influence of the seasons on mortality. In pursuing this inquiry an unexpected fact has come out on phthisis. By these calculations, opposed to others made in the metropolis, it seems fully ascertained that consumption is more frequent in the south-west of Devonshire than in London. It may be proper to state, that the estimate is not taken from strangers who carry the disease to this part, but from the registry of a Dispensary at Plymouth.

In the preceding volume of the *Med. & Phys. Journ.* the writer of this annual Report has endeavoured to point out some of the benefits that would arise from a *medical topography* of the British Isles. Dr. Woolcombe has furnished additional arguments in support of such a design, by the development of many interesting local facts. There can be but little doubt that every portion of the united kingdom has some local peculiarities, of importance, probably, to the extensive views it is essential that the *science* of medicine should take. How easily would a knowledge of these local peculiarities be collected, if medical practitioners, wherever they are stationed, did inquire into the facts by which they are surrounded, and, through the medium of some channel, from time to time, give their discoveries to the public.

Among other advantages that arise from investigations such as Dr. Woolcombe's, those are not the least, perhaps, which tend to correct popular opinions on the subject of seasons as affecting the health, and which are frequently the reverse of truth. In observations on weather and diseases from 1751 to 1757, Doctor Fothergill asserted, that excess of wet, with moderate warmth, is not so dangerous to health, as a dry and cold season. The truth of this has been demonstrated by Dr. Heberden. No fact is, perhaps, better established than that a severely cold and dry atmosphere, occasions a greater mortality in this climate, than an open winter with wet. From documents collected in various parts of Britain, Dr. Woolcombe shews, that the relative healthiness of the months of the year follow this order;—July the most healthy, then August, June, October,

October, November, May, December, April, February, January; and, the most productive of disease, March*.

In the department of Pharmacy and the Materia Medica the present year affords but little of novelty. The College of Physicians of London, employed on a new edition of their Pharmacopœia, have dispersed among the fellows and licentiates, two specimens of their intended publication. The materia medica appears to have received an addition of some value in the *Radix Rhataniæ,* a substance long known to the manufacturers of Port wine. This drug is the production of Peru, and is probably the root of the Cinchona cordifolia. It is described as externally resembling the rubia tinctorum, to the taste being aromatic, bitter, and very astringent; its infusion or decoction, turns black with sulphate of iron, and precipitates tannin. The principal virtues appear to reside in the cortical part of the root, which is thick and resinous. An opinion prevails that the substance, sold in the shops under the name of foreign extract of bark, is made from the Rhatania. The first to blazon the virtues of a new remedy, are those who deal in it; but the reasons for suspecting their evidence, are very obvious. In every instance their reports should be taken with great caution. On safer evidence, however, than the assertions of traders of any description, it is known that this substance is a powerful tonic. In debility of the digestive organs, in chronic rheumatism, fluor albus, and in intermittent fever, it has been employed with very good effect. While given in doses similar to cinchona, it has the advantage of being only one third the price of that substance.

To the practical part of botany, and to those higher and

* The local as well as temporary history of health, as affected by the seasons, should be fully understood: the description of Celsus, compared with the above, will put this in a strong light. On the shores of the Mediterranean he said, *saluberimum ver est: proxima deinde ab hoc, hiems: periculosior æstas: autumnus longe periculosissimus.* And he explains why the autumn was most sickly. *Ex tempestatibus vero optimæ æquales sunt, sive frigidæ, sive calidæ: pessimæ, quæ variant maxime. Quo fit, ut autumnus plurimos opprimut. Nam fere meridianis temporibus calor; nocturnis atque matutinis, simulque etiam vespertinis, frigus est.* There is no question but this has given a bias to the opinions of the faculty in this country, for a long period, and induced them to assert, and probably to believe, that the autumn was here as in Italy, the most sickly season. We observe for ourselves, and discover that what might be very true of Italy, cannot be applied to Britain.

more elaborate views that raise the practice to the dignity of science by investigating the relations, analogies, and functions of vegetable life, the art of medicine is much indebted. And in a former Report, an effort was made to impress the practitioner, and particularly the student, with a sense of the benefit that would arise from a due attention to this subject. In the natural botanical analogies, it was presumed there was a clue that led to the knowledge of a considerable extension of medicinal properties in plants. Simple and obvious as this principle appears, it is accompanied with circumstances that should induce a cautious study of the subject; for it will be found that many individuals of the same family possess properties widely differing from their congeners, and even distinct parts of the same plant afford substances of very opposite qualities. The leaves of the Jatropha manihot are employed as a common esculent; but its root secretes a virulent poison;—the genus Laurus associates in the same family the Cinnamon and Cassia with the poisonous L. caustica;—the Convolvulus batate (Spanish potatoe) is joined to the Jalapa and Scammony, and the common potatoe (solanum tuberosum) is found among the Nightshades. These facts do not subvert the principle drawn from botanical analogy, yet as they are of no infrequent occurrence, they forcibly indicate the necessity of cautious inquiry. But it is not from eliciting the qualities of plants only, that Botany holds an elevated station among the elementary sciences that form the medical character; the structure, the functions, and the diseases of vegetables, and the many other analogies they have with the operations of animal life, form so near a connection with anatomy, physiology, and the institutes of medicine, that they mutually illustrate each other. On the continent this seems to be fully understood; and Professor Willedenow has ingeniously arranged in a portable form, the known facts belonging to the subject. From the oration before the Medical Society of London, at their anniversary on the 8th of March, it may be seen, however, that the subject is not altogether unnoticed here. In this oration Mr. Good gave a brief survey of the general structure and physiology of plants compared with those of animals, and the reciprocal conversions of animal and vegetable matter. The nature of the subject brought together a great number of interesting and curious facts; and the student who desires to pursue the inquiry, is much indebted to the ingenious ous author for having compressed into a small pamphlet the spirit of many volumes *.

To bring the practice of medicine and surgery to the highest possible state of perfection, has been often, if not always, the aim of professional study. And as there are but few practitioners, who have not, in some moment of self-complacency, believed they had made discoveries that would be serviceable to the public, it has necessarily followed that the practical department of medical literature has teemed with the labours of a multitude that sought the "bubble Reputation." In the year 1808 we have seen this desire extend from the science of Willan, downward, with a long interval, to an improved system of medicine by an ingenious Colonel. It will not, perhaps, be proper to look very closely into the causes that induce to publication; but rather to admit, with prudent silence, the benefits that science directly or indirectly receives. The learned physician, just mentioned as the strongest possible contrast to the mob of authorlings, has long been employed on the investigation of an obscure and difficult class of diseases. He found the history of cutaneous complaints involved in confusion; one description serving for a multitude of synonyma, or one synonym often indicating different diseases. Arrangements, when they were attempted, were erected on uncertain and variable data; and the suggestions of fancy supplied the facts of nature. To this chaos Dr. Willan applied the principles that have guided the classifications of Natural History. He selected the least mutable of the appearances that occur in cutaneous diseases; these he defined with scrupulous exactness, and employed them as the foundation of seven orders, that were to comprehend all the genera, species, and varieties of morbid affections that happen to the skin. These orders were characterised by

* Oxygen is the principal stimulus in the germination of plants, and affords a test of remaining vitality in torpid seeds. A torpid seed, destitute of all power to vegetate in any other substance, if steeped in a diluted solution of oxygenated muriatic acid, at a temperature of 46° or 48° of Fahren. if any portion of vitality remains, will germinate in a few hours; and if then planted in its appropriate soil, will grow with as much speed and vigor as if it had not evinced torpidity. We wish to extend the publicity of this fact from the utility connected with it. As applying particularly to the torpid state of seeds brought from distant countries, it may obviate the disappointment of many who are anxious to extend botanical knowledge, by exciting the latent spark of vitality where it was believed to be extinguished. Possibly the application of this acid, in some particular state of dilution, to growing plants, may produce unexpected effects.

by the different appearances of papulæ, scales, rashes, vesicles, pustules, tubercles, and maculæ. This great work has advanced slowly, because, perhaps, rapidity was incompatible with correctness; or impossible where the subject was to receive the last touches of elaborate finishing. Two Orders, and a part of a third have been some time published; and at length a fourth fasciculus, compleating the third Order, and comprehending the fourth, has appeared. With this Order ends the first volume; and it is but common justice to say it is executed with a correct attention to nature, with a spirit of investigation and comprehensive research, that are highly creditable to the author; and that it is accompanied throughout with plates that cannot fail materially to illustrate the description of morbid appearances.

Among the diseases that have been distinguished for the difficulty with which remedies act upon them, Diabetes has been conspicuous. Before Dr. Rollo's observations on this disease were made public, it was treated with astringents and tonics; he attempted, and in some instances succeeded, to cure it by animalizing the system.* The present year has produced another method, perfectly distinct from either of the former, and, it is said, still more successful. Mr. Watt, an ingenious surgeon at Paisley, disappointed in his attempts

* Mr. Watt has very ingeniously made his system of abstinence quadrate with the use of animal food, as employed by Dr. Rollo. "That abstinence to a considerable extent, he observes, obtained in Dr. Rollo's cases, may be inferred from the nature of the diet to which his patients were restricted:—animal food alone, and that as rancid as possible. Their constant deviations, in spite of every remonstrance, are convincing proofs that they soon acquired a dislike to it; and their being denied every thing else might be the means of enforcing, though inadvertently, a sufficient degree of abstinence. The sickness produced by the hepatised ammonia, must also have contributed to the same end." The instability of medical hypothesis has always been a subject of regret. But in few instances has a theory of great ingenuity, remarkable simplicity, and of apparently easy adaptation to practice, been so suddenly subverted as Dr. Rollo's has been by Mr. Watt. The use of animal food of such a kind, and in such a state as to be highly disgusting, combined with restriction from all other nutriment, must virtually be the same as a system of abstinence.

The leading points in Mr. Watt's treatment of Diabetes, are—"1st. To reduce the quantity of blood already in the system.—2d. To diminish the supply of new materials.—And, 3d, To afford a certain proportion of stimuli. This is accomplished, 1st. by venesection, or any other competent evacuation.—2d. By abstinence.—3d. By blistering, mercury, &c. The effect of this treatment on the system is, increased reaction, indicated by a degree of fever, and terminating in the restoration of all the excretions." The ingenious author has denominated this, the treatment, the renovating process, and the cure.

attempts to relieve his diabetic patients by former methods, employed abstinence and venesection. The limits of this Report do not admit of a detailed view of the train of reasoning employed by Mr. Watt, to justify his adopting these bold measures, or of a particular statement of the facts that induced him to employ a plan that would, it was believed by competent judges, be suddenly fatal in a disease of such apparent debility. Instead of this fatal termination taking place, however, patients who lost from their system very large quantities of blood, 108 ounces in a fortnight, had their disease suddenly subdued, while the energies of the frame increased in proportion to the frequency of evacuation. The singular change produced on the circulating fluid by these evacuations deserves particular notice. The first drawn blood was thin, abounded with serum, had little coagulum, and that without firmness and buffy coat. As the venesections were repeated, the blood assumed an inflammatory character, had an increased quantity of crassamentum, and separated a coagulum, tenacious, buffy, and cupped, till contracted on its upper surface to the size of a shilling. The striking character of these facts, and the impetus they will give to the tide of opinion that is strongly setting against the stimulating therapeutica of the Brunonian school, will claim for them the most serious attention. But while the originality of Mr. Watt's pathology, the ingenuity of his remarks, and his novel treatment of several diseases, for he has extended his researches to asthma, cholera, colic, consumption, chorea, and plethora, must be allowed; it is experience alone that can determine their value.

If the bold deviations of Mr. Watt have not pervaded every publication of the year, yet several are distinguished by a patient investigation of the progress, symptoms, and cure of disease. Dr. Badham's observations on inflammatory affections of the mucous membrane of the bronchiæ, Dr. Davis on carditis, and Dr. Cheyne on hydrocephalus acutus, deserve attention as practical works that will afford considerable information.

It seems a singular incident in the history of medicine, that the most dreadful malady to which human nature is liable should have occurred so frequently in this year, without having occasioned any separate publication of importance. In this Retrospect for last year, the investigations of Dr. Bardsley on *rabies canina* were mentioned with an approbation to which they were entitled; and calculated, it was hoped, to excite public attention to the means he proposed for extirpating, or checking at least,

 the the spread of this disease. The events of another year have fully shown how essential to the security of society some means for the prevention of this complaint are become. In the short period of a few months many fatal cases have happened; and this frequent recurrence has at length produced so just a sense of the danger, as to call forth the direct interposition of the legislature. In the latter end of the year the Secretary of State directed the College of Physicians of London to inquire into the actual prevalence of rabies within the metropolis, and its vicinity, with a view to its prevention or cure. This requisition occasioned an advertisement from the College Register, requesting practitioners to communicate such cases as had fallen under their personal observation, and in which hydrophobia had actually taken place; together with a statement of leading facts sufficient to identify the disease. It is generally understood that this public notice occasioned a large mass of materials to be transmitted to the College. Beside the materials thus collected, and which have in no part been made public, several well discriminated cases have been printed, in which the progress the symptoms has been detailed with a precision that marks scientific investigation, and it is feared has also ascertained the inefficacy of every known remedy. With this dread of rabies, which has spread very generally throughout the kingdom, much false alarm has doubtless been mixed, and many dogs have been sacrificed without the real nature of their disease being known. It is not easy to conceive any circumstance of existence more dreadful than the distress that must arise after being bitten by a dog, when the destruction of the animal has precluded the possibility of ascertaining his malady. Probably under this mental calamity, nervous symptoms have arisen imitating the specific hydrophobia so nearly, as with small aid from the imagination, to be pronounced that disease: and the symptoms thus excited, having worn out by time, or being subdued by medicine, have afforded occasional instances of cured hydrophobia. It is certain that a want of discrimination among the diseases of dogs, has occasioned infinite distress, and probably very serious consequences. Dr. Clarke of Nottingham has described a disease in this animal, which occurred in that town and neighbourhood in August and September; and might have been mistaken for rabies. Several persons were bitten, but no instance of hydrophobia ensued. In this affection the dogs were suddenly attacked with vertigo, turning round, staggering, and biting biting at objects in their way, and then unexpectedly falling down dead: and often in two or three hours after the first seizure. Some have looked dull in the eye, having a frothy saliva in the mouth: in them the tartrite of antimony removed the disease. These complaints prevailed most among dogs exposed to the temporary influence of fire; and an instance occurs of a dog being suddenly seized with phrenitis from this cause.

The frequent occurrence of bites from dogs under suspicious circumstances not being followed by any disease, and the appearance of a malady said to be produced by the bite of a mad animal, when it was known that no application of the rabid virus had taken place, occasioned doubts whether a specific disease, arising from a poison sui generis, infused by the bite of a rabid animal only, ever occurs: or whether the symptoms said to arise from this source are not produced by affections of the nervous system, induced by terror, occasioned by a lesion of nervous fibre, or connected with some inflammatory state of the œsophagus or stomach. That a morbid state of the system sometimes happens, resembling hydrophobia so nearly as to be called by nosologists hydrophobia spontanea, cannot be doubted; but this differs in several of its phænomena from the specific disease. In its course and symptoms it so nearly resembles tetanus, that it may be considered as a variety of that affection. It has followed the bite of a dog, and it has occurred from wounds in various parts, certainly unconnected with the bite of any animal; it is often violent in its symptoms, sometimes terminates in death, but commonly in recovery. In many parts of its character it differs from the disease so clearly described by Drs. Pinckard and Powell. In one particular there is a marked dissimilarity: the specific disease is always fatal, the spontaneous is generally cured. The following case comes so near to the specific disease, that it may serve to illustrate the opinion that all the cured cases have been simple hydrophobia. An athletic young man in high health, on a Saturday afternoon in the month of July, was attacked with uneasy feelings about the neck and muscles employed in deglutition: swallowing was rather impeded, particularly of fluids. In the evening the symptoms were so much increased as to require medical assistance. Swallowing was then become more difficult, the pulse hard, and there was an efflorescence about the fauces, that indicated the approach of active inflammation. Early on Sunday morning the inflammatory state of the fauces had so far subsided as to do away the apprehension of angina angina inflammatoria. The pulse had now increased in rapidity; there was a wild quickness in the countenance; some slight involuntary actions of the muscles appeared about the upper part of the frame, that could hardly be deemed convulsive; and the difficulty of swallowing fluids was alarmingly great. Whenever an attempt was made to force down liquids, it was only effected with reluctance, hesitation, hurry, and confusion; accompanied with inexpressible distress, and spasms that spread from the muscles about the throat and neck to those of the shoulders and back. So strongly marked was this dread of fluids, for the sight of them produced disturbance, that it became the principal object of attention; indeed, so slight were all the other symptoms, and so quiescent, except when fully excited by attempts to drink, that probably at this period of the disease, 19 hours after its visible accession, they would not have been the subject of medical investigation. Inquiries were now made, with the caution that should prevent its concealment if it had happened, whether the patient within a few weeks, or at any preceding time, had been bitten by a dog or other animal. It was positively denied by his parents and himself, persons of veracity and good sense, that any such circumstance had occurred; and from every inquiry it became certain that no such accident had happened. At eight on the evening of this day, the dread of fluids had risen to a most alarming point; convulsive spasms, particularly of the muscles of the back curving the spine backward, were become frequent, but were always excited by attempts to drink. The pulse was now extremely rapid, the face flushed, temperature high, and the skin covered with profuse perspiration. To these were added, a rigidity of the lower jaw, which impeded its motions without closing the teeth. It was now stated by his parents, after reiterated inquiries whether accidents of any kind had happened to him, that three weeks before he had ran the iron tooth of a garden rake into the ball of the great toe; that for a few days it was painful, but got well without assistance. It may be remarked, that this was the unbiassed relation of the parents, for no assurance or reasoning could persuade them that their son's disease arose from so trifling an accident. On examining the toe, a slight scar was seen where the iron had entered; but there was no other appearance of local disease. On Monday morning every symptom had become more intense, the convulsions were more frequent and violent; it was impossible to get down any fluid; the pulse was 150, with a disposition position to delirium rather than its actual existence, except during the spasms, when it required much force to keep the patient in bed; and this was resisted with incoherent expressions, threats, and fierce ravings. In the afternoon of this day, after a succession of frightful convulsions, he expired, forty-eight hours after the appearance of the disease.

This history of a case of tetanus, accompanied throughout all its stages with a strongly marked hydrophobia, had it followed the bite of any animal, must have been attributed to the action of the rabid virus. Its rapid progress to a fatal termination, also brings it nearer to the specific disease than most other recorded instances of simple hydrophobia. The apparent approximation of many diseases, specifically very different, has led to some fatal mistakes, and to more impositions. How often have consumptions been cured that were only catarrhal affections; and the disease which is the immediate subject of these remarks, has, in its simple form, been subdued again and again, sometimes by substances absolutely inert, at others by the powerful articles of the materia medica; while the specific affection resists the utmost energy of medical art. A little reflection will prevent practitioners having recourse to methods, in themselves generally empirical, and which have failed constantly when the disease has been really what its name implied. But the scientific physician will not merely reject what experience has found to be inefficient, he will endeavour, by a close study of the disease, to find fresh expedients, and to discover more successful modes of treatment.

The heat in the month of July of this year far exceeded that of any other reported period; but it does not seem directly to have occasioned any increase of disease. Many rumours were promulgated of its suddenly fatal effects, but very few of these were true; or were rather exaggerated accounts of common occurrences, referred to this high temperature as their cause. It is very probable, however, that some persons employed in the fields suffered something like a coup de soliel; but generally during this hot weather the state of health was more perfect than in seasons when such deviations have not been observed. Circumstances occurred in the autumn and winter, which indicated, however, that the influence of this heat had been felt throughout the kingdom; and that a very extensive epidemic state of the atmosphere probably resulted from it. Over most parts of the country, but particularly in the marshy districts, in the autumn autumn of the year, after the subsidence of this excessive heat, intermittents became frequent, to a degree seldom before experienced; and this disposition in the air continued for many months, and during the cold and frost of winter. Even in London tertians were often met with, though in several instances they were traced to sources very distant from the metropolis. If the cause of intermittents is some marshy exhalation, it is a question, how long the appearance of the effect may be protracted after the application of the cause? A family whose summer residence is generally, and was during the heats of this summer and to the end of November, in the midst of the fenny parts of the Isle of Ely, had, after their return to town in different years, several individuals at uncertain periods, seized with intermittents. Their town residence is far removed from every local circumstance that can be supposed a cause of this disease, being in a high and dry part near to Grosvenor Square. The disease itself has taken in them the form of tertian, and has had an obstinate continuance. The last case occurred in April 1809 to a young woman who left the country in November 1808. Observations relative to the extent of the interval that may take place, between the application of the exciting cause and the appearance of ague, are not yet sufficiently numerous to warrant any positive conclusion. It is probable, however, that the disease may not appear till a considerable time after exposure to the cause, and at a considerable distance from the place where it was contracted.

Among the evils of protracted war must be enumerated the origin of new diseases, or at least the aggravation of those already existing, to a degree of malignity that gives them a new character. The campaign in Egypt was connected with the spread of an ophthalmy of unknown virulence, and propagated by infection. The extensive progress of this disease, and the multitude of cases that terminated in the destruction of the organ of vision, excited the attention of the profession, and many ingenious disquisitions have been published. In the present year the remarks of Mr. Ware on the purulent ophthalmy that has been epidemic in this country, deserve attention. Connected with this is an anonymous pamphlet, with the quaint title of "Identities ascertained;" in which the writer professes to illustrate the opinion of Mr. Ware respecting the sameness of infection in gonorrhœa and the ophthalmy of Egypt. There has also been published an ingenious practical treatise on the Egyptian ophthalmy, by Mr. Thomas; and and the first part of a work of considerable importance on the morbid anatomy of the eye, with plates, by Mr. Wardrop. Considerable attention has likewise been given to other branches of Surgery. The operation of lithotomy in particular has been investigated by Mr. Allan of Edinburgh, who has endeavoured to demonstrate the dangers of operating with the gorget, and to point out the superiority of the more simple operation by the knife and staff. Dr. Thompson, also of Edinburgh, has re-published Dr. James Douglas's appendix to his history of the lateral operation, and of the other original papers relative to Mr. Cheselden's invention and improvement of that operation. To this Dr. T. has added a proposal for a new method of cutting for the stone; which consists in the use of a scalpel and staff in a way somewhat different to Mr. Allan's. Mr. Simmons, of Manchester, has published a small pamphlet on the same subject, the object of which is to point out the advantages of a pure atmosphere to patients who have undergone the operation of lithotomy; and to describe, what he conceives to be some improvement in that operation. The calculus of animals has been so often tortured by chemical analysis, as scarcely to admit the hope of farther illustration. But of what avail is this? The knowledge of the chemist has not furnished the means of cure; and the painful and hazardous operation just noticed, is now as much a resource, as before it was known of what the calculus of the urinary bladder was compounded. We lament the fact without meaning to detract from the merit of the chemist, and would be well pleased to have understood, that after the examination of 150 human calculi, Mr. Brande had discovered something to alleviate the sufferings of human nature, as well as that a small number only of the 150 were composed of pure uric acid; and that in the calculus of the horse, the ox, the sheep, the dog, the hog, and the rabbit, he found carbonate of lime. There is, however, in Mr. Brande's experiments, conjoined with Mr. Home's observations, a negative merit. They shew why the supposed solvents do not effect a cure, and also why, in many instances, they increase the malady; and that in others, where it has been believed they had effected a cure, the suspension of symptoms had arisen from a very different cause, *an enlargement of the posterior lobe of the prostate gland.* There is no disease that afflicts man more connected with positive corporeal suffering than stone; but there are many that put life in greater hazard, and when understood by the patient, impress with stronger mental apprehensions.

Aneurism

Aneurism presents an instance. The mind must be painfully harrassed when it is known that any sudden exertion, or the next throb of the heart, may bring instantaneous death. But the fears of the patient may be much lessened by understanding that all varieties of aneurism are not equally hazardous or incurable. This fact has been long known to surgeons, but it has been remarked with a nicer discrimination since the year 1804, when Professor Scarpa of Pavia, in a splendid work, pointed out the shades of difference that exist among the cases of this disease, connected with their natural history and pathology, in a way that distinctly marked the peculiarities of every species and variety. The merit of this work is not that of communicating original discovery, or of presenting ingenious novelties; but it has the fair character of giving to the profession an elaborate history of the state of knowledge on the subject of aneurism, by a perfect master of the subject. And it is satisfactory to observe that an English translation of it, by Mr. Wishart, is added to the medical literature of this country; though it must be lamented, that in a nation like this, motives of œconomy should have compelled the translator to omit the exquisite plates of Professor Scarpa.

It has been observed until observation has become trite, that the study of medical jurisprudence has been neglected in Britain to a degree passing belief, but from experience of the fact. At length the profession seems to be aroused to a sense of its utility by an occurrence that took place in the autumn of this year. A young woman died at Liverpool under circumstances so mysterious as to induce a legal inquiry. The body was examined by several physicians and surgeons of eminence, whose evidence went to prove that she died by poison: but it did not appear that any remains of that poison were detected in the stomach, or that the symptoms during life, were precisely such as have been *commonly* understood to characterize the action of a virulent irritative poison. Secondary to this, it was endeavoured to be proved that this person had been delivered of a child very shortly before her death, because the uterus was found distended, and had marks of the recent attachment of a placenta. These opinions were impugned in court by one physician, whose remarks so far raised doubts in the jury, that they acquitted the accused person. It will be readily seen that this case involves many difficulties, and calls into action a variety of contending physiological opinions; difficulties hard to be solved, and opinions, that even the weight of authority cannot reconcile. The unexpected turn of the trial occasioned sioned further medical investigation, and the case has been re-examined in a variety of pamphlets of various merit. One useful consequence will result from this controversy: it must induce the faculty seriously to reflect that they stand between the law and the accused; and that on their evidence an innocent person may be convicted, or a villain escape punishment. And if this investigation, which has been carried on with a warmth not ill calculated to make an impression, should raise in the faculty a proper sense of the important station they fill in society, and the influence their opinions have on the fate of their fellow creatures; it will also lead them to feel that they cannot conscientiously decide in intricate cases, without laborious research and profound investigation.

With a view to the credit of the profession under every trying circumstance, it has always been a primary consideration with the writer of this annual *Retrospect*, to impress the minds of students, at least, with a full conviction that the *science of Medicine* is founded on an exact acquaintance with the numerous facts that are spread throughout Nature; and that their knowledge will be superficial or profound in a ratio agreeing with the proportion in which these facts are ascertained, arranged, and understood. As far as the learning of antiquity has been transmitted to us, we have seen that on subjects least allied to nature and the properties of life, that learning has been most perfect. But from the revival of literature there has been a gradual approach to a true history of the visible creation; and the practice of medicine has been benefited by this direction of the human mind, in a proportion greater perhaps than any other branch of knowledge. Within the limits assigned to this Report it would be impracticable, if it were proper, to point out the various circumstances connected with the elucidation the science of life has undergone in this period; or to do even a slight justice to individuals who have pushed on discovery in every direction allied with this interesting subject, to a point, we hope, of scientific certainty. Yet among the causes that in modern times have particularly contributed to disseminate a knowledge of nature, we cannot forbear to observe, that the institution of philosophical societies, the occasional publication of materials collected by them, and the establishment of scientific journals, have been efficient in an eminent degree. By this means a facility of communication has been afforded, as well as an asylum for fugitive remarks, suggestions, and facts, that would otherwise have have perished in the progress of time. Within the immediately preceding period of twenty-five or thirty years, on the continent of Europe, in the British Islands, in America, and in India, a continued sense of the public utility of this periodical communication of the progress of science, has been clearly evinced by the encouragement it has received. A writer of peculiar energy has observed, that "an age of war is not often an age of learning: the tumult and anxiety of military preparations seldom leave attention vacant to the silent progress of study, and the placid conquests of investigation." But we have now to lament that a warfare, rancorous beyond any precedent in civilized life, has closed the course of useful information, forbidden the intercourse of nations, destroyed or suspended the mutual advantages that arise from the collision of opinion, and the union of inquiry among men destined to similar pursuits, and labouring in the vineyard of science. With the whole of the continent of Europe, and with America, the intercourse of the last eighteen months has been so difficult, uncertain, and inefficient, that we have been compelled, in giving this view of the progress of the medical art, to collect the principal part of the materials from resources, in a great measure, confined to the British Islands. But if we regret this abridgement of the means of information, we cannot but feel a proud hope, that the energies of the British nation may become more elastic by compression; and as the power of France to shut the continent extends, that the intellect with which this happy "nook of earth" is blessed, may with tenfold activity be exerted, to the elucidation of science and philosophy, to the detection of error, and to the promulgation of TRUTH.

*Princes Street, Cavendish Square,*
*June,* 1809.

